# Natural and Synthetic Derivatives of Hydroxycinnamic Acid Modulating the Pathological Transformation of Amyloidogenic Proteins

**DOI:** 10.3390/molecules25204647

**Published:** 2020-10-12

**Authors:** Vladimir I. Muronetz, Kseniya Barinova, Sofia Kudryavtseva, Maria Medvedeva, Aleksandra Melnikova, Irina Sevostyanova, Pavel Semenyuk, Yulia Stroylova, Matej Sova

**Affiliations:** 1 Belozersky Institute of Physico-Chemical Biology, Lomonosov Moscow State University, 119992 Moscow, Russia; Kmerkushina@gmail.com (K.B.); alksmelnikova@gmail.com (A.M.); seva2@mail.ru (I.S.); psemenyuk@belozersky.msu.ru (P.S.); ylkamail@gmail.com (Y.S.); 2 Faculty of Bioengineering and Bioinformatics, Lomonosov Moscow State University, 119234 Moscow, Russia; sofiia.kudriavtceva@gmail.com (S.K.); maryshick@mail.ru (M.M.); 3 Institute of Molecular Medicine, Sechenov First Moscow State Medical University Trubetskaya St. 8, Bldg. 2, 119991 Moscow, Russia; 4 Faculty of Pharmacy, University of Ljubljana, Aškerčeva 7, 1000 Ljubljana, Slovenia; matej.sova@ffa.uni-lj.si

**Keywords:** neurodegenerative diseases, curcumin, derivatives of hydroxycinnamic acid, amyloid, α-synuclein, prion protein, amyloid fibrils, prevention of amyloid transformation, Parkinson′s disease

## Abstract

This review presents the main properties of hydroxycinnamic acid (HCA) derivatives and their potential application as agents for the prevention and treatment of neurodegenerative diseases. It is partially focused on the successful use of these compounds as inhibitors of amyloidogenic transformation of proteins. Firstly, the prerequisites for the emergence of interest in HCA derivatives, including natural compounds, are described. A separate section is devoted to synthesis and properties of HCA derivatives. Then, the results of molecular modeling of HCA derivatives with prion protein as well as with α-synuclein fibrils are summarized, followed by detailed analysis of the experiments on the effect of natural and synthetic HCA derivatives, as well as structurally similar phenylacetic and benzoic acid derivatives, on the pathological transformation of prion protein and α-synuclein. The ability of HCA derivatives to prevent amyloid transformation of some amyloidogenic proteins, and their presence not only in food products but also as natural metabolites in human blood and tissues, makes them promising for the prevention and treatment of neurodegenerative diseases of amyloid nature.

## 1. Introduction

Such socially significant diseases as Alzheimer’s, Parkinson’s, Huntington’s, prion diseases of humans and other mammals, and other neurodegenerative diseases, are associated with aggregation of disease-specific proteins, which leads to the formation of amyloid oligomers and fibrils [1,2]. These protein species are thought to be toxic to cells through a myriad of mechanisms, including active cell-to-cell spreading [3,4,5]. One of the directions in which attempts are made to create therapeutics is a search for compounds preventing formation of pathological amyloids or destabilizing preformed amyloid fibrils and oligomers. Particular success was obtained by the application of monoclonal antibodies as highly targeted drugs, offering the possibility to not only target a specific protein, but also a specific conformation. Such antibodies are currently under clinical trials for Alzheimer’s disease [6]. Information is available on anti-amyloid activity of other macromolecular compounds including synthetic ones, such as polyelectrolytes, dendrimers, and others [7,8,9,10,11]. Ultimately, the use of macromolecular compounds interacting with target proteins at multiple sites, forming different types of bonds, increases recognition specificity. Nevertheless, the problem of passage through the blood–brain barrier limits the potential application of macromolecular compounds. Furthermore, the high cost associated with the production of the most promising compounds, monoclonal antibodies, does not allow us to rely on their widespread use. For the aforementioned reasons, an effort has been put into the search for small molecule compounds with anti-amyloid and anti-aggregation activities capable of transporting across the blood–brain barrier. Various small molecule anti-amyloid compounds were reviewed recently [12,13]. In this review article, we will highlight the discussion of recent data on hydroxycinnamic acid (HCA) derivatives, including the results obtained recently in our work.

In case of prion protein, interest in HCA derivatives as anti-amyloid compounds stems from their structural similarity to a “half” of the curcumin molecule. Curcumin is a natural polyphenolic compound with various prophylactic and medicinal properties. Curcumin has a wide spectrum of biological activities, including antioxidant, anti-inflammatory, antitumor, hepatoprotective, antifungal activities, inhibition of a number of enzymes, iron chelation and neuroprotective activity [14,15,16,17,18]. Curcumin is also known to inhibit pathological aggregation of some amyloidogenic proteins in vitro, for example, prion protein (PrP) [19,20], α-synuclein [21,22,23], and β-amyloid peptide [24,25]. Moreover, curcumin was shown to disaggregate preformed amyloid fibrils. However, despite the huge amount of studies on the effect of curcumin on various pathological processes, the available information is, in general, rather contradictory. Some researchers believe that the idea of curcumin being a promising lead that can potentially cure many diseases is vastly exaggerated [26]. Conflicting information may be accumulating due to the low solubility of curcumin in aqueous solutions, the poor bioavailability of this compound, and therefore, the inability to accurately calculate concentrations of this substance, producing various effects. HCA derivatives, on the one hand, are structurally similar to a curcumin molecule, more precisely, half its’ molecule, and on the other hand, are readily soluble in water, thus being deprived of these drawbacks. Another advantage of at least some HCA derivatives is their natural origin. They are not only present in various foods and medicinal plants, but are also found in human blood, meaning they are natural products of metabolism. This circumstance reduces the risks associated with their potential use as drugs and prophylactic remedies.

In this review, we will discuss general information about HCA derivatives, and then present the main approaches for the production of various natural and synthetic HCA derivatives, which are useful for the investigation of mechanisms of their interaction with target proteins. Then we will discuss the results of molecular modeling of HCA derivatives’ binding to known structures of monomers and fibrils of amyloidogenic proteins. The main focus of the discussion will be on the influence of HCA derivatives and structurally close compounds on the amyloid transformation of PrP and α-synuclein.

## 2. Naturally Occurring Hydroxycinnamic Acids and Their Derivatives

**Figure 1:**
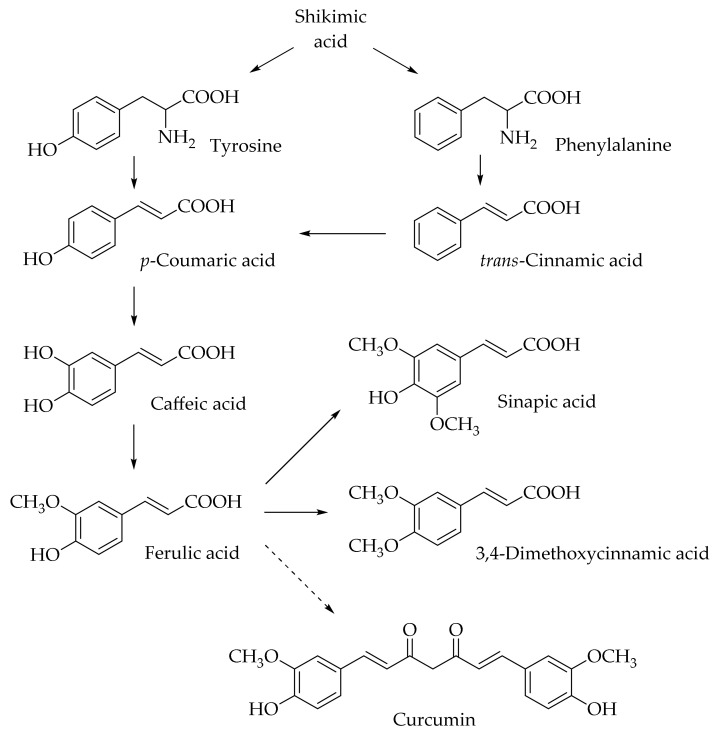
Biosynthesis and structures of naturally occurring hydroxycinnamic acids and curcumin.

Hydroxycinnamic acids (HCAs) are a class of phenolic compounds whose characteristic structural feature is the phenolic ring and a radical containing a carboxyl group (Figure 1). These compounds differ from each other by substituents on the phenolic ring. Naturally occurring HCAs, namely ferulic, *para*-coumaric, caffeic, sinapic acids and 3,4-dimethoxycinnamic acid (3,4-DMCA) are present in significant quantities in the cell wall of plants. In plants, these compounds are formed through the mevalonate-shikimate biosynthesis pathway, which allows plants to synthesize essential amino acids from the primary metabolites phosphoenolpyruvate and erythrose-4-phosphate [27]. Upon deamination of phenylalanine and tyrosine, *trans*-cinnamic and coumaric acids, respectively, are synthesized. The routes of synthesis and further conversions of HCAs are shown in Figure 1. Some of them are of particular interest since they display various confirmed biological activities and are widespread in plants.

HCAs are ubiquitous in foods of plant origin and can make up about a third of the phenolic compounds in our diet [28]. The predominant HCA in most fruits is caffeic acid, accounting for more than 75% of the total amount of HCA derivatives found in plums, apples, apricots, blueberries, and tomatoes [29]. However, *p*-coumaric acid is predominant in citrus fruits and pineapples [30]. Generally, HCAs are present in the bound form and are rarely found in a free form. Processing of the fruits and vegetables (freezing, sterilization, and fermentation) promotes the formation of free HCAs in plant products. The most common water-soluble derivatives of HCAs are esters between the carboxyl group of the phenolic acid and one of the alcohol groups of the organic compound, for example, chlorogenic acids (esters of HCAs and quinic acid), and glucosides with a glycosidic bond with the hydroxyl group of the HCA derivative (for example, *p*-coumaric acid *O*-glucoside).

Ferulic acid is one of the most abundant phenolic compounds in the cell wall of plants. Ferulic acid can be found in all living species, from bacteria to humans. It can be absorbed in the small intestine and excreted in the urine. The content of ferulic acid in plants is considerable, varying from 5 g/kg in a wheat bran to 9 g/kg in the pulp of a sugar beet and 50 g/kg in the kernel of a corn [31]. It is found mainly in seeds and leaves, both in free form and covalently linked to lignin and other biopolymers. It is usually found in the form of esters, cross-linked with polysaccharides in the cell wall, such as arabinoxylans in herbs, pectin in spinach and sugar beets, and xyloglucans in bamboo. Crosslinking with proteins is also possible. Ferulic acid is usually found in the highest concentration in grains and has also been found in plant foods such as burdock, eggplant, grapefruits, oranges, soybean, and bamboo shoots [32]. Caffeic acid was found but not quantified in significant amounts in foods such as cereals and cereal products, arabica coffee, fruits, plants, oils, grapes, and tea [33]. Sinapic acid is found in dietary plants such as rye, citrus and berry fruits, vegetables, cereals, and oilseed crops [34,35,36].

The daily intake of caffeic and ferulic acids in humans is estimated to be 500–1000 mg from fruits, vegetables, grains, bran, beer, and coffee [37]. HCAs in a free form are absorbed by monocarboxylic acid transporters in the mucous membrane of the gastrointestinal tract. The absorption efficiency largely depends on the affinity of the compounds for monocarboxylic acid transporters, and when HCAs were administered to rats, it was shown that the affinity scale grows in the order of caffeic acid < *p*-coumaric acid = ferulic acid [38]. The peak plasma content for ferulic acid is reached 5–10 min after oral administration [38,39].

3,4-DMCA and its derivatives, which are components of black coffee, and especially green coffee beans, are noteworthy [40,41,42,43,44]. These compounds are bioavailable and active in the body through the use of coffee [45,46].

In fact, the plant *Coffea canephora var. robusta* contains a higher level of 3,4-DMCA (X ±SD: 0.433 ± 0.15, in the range of 0.237 to 0.691 g/kg) than *Coffea arabica* (X ±SD: 0.059 ± 0.03, in the range from 0.016 to 0.095 g/kg) [47].

3,4-DMCA is of particular interest because it appears in large quantities (~380 nM for 60 min) in human blood plasma after coffee uptake, despite the low concentration in the coffee extract. Ferulic acid also appears in plasma in a free form after coffee consumption. Interestingly, 3,4-DMCA constitutes only 3% of the ferulic acid content in coffee extract, but its concentration reaches 2.4-times higher than that of the ferulic acid in blood plasma after drinking coffee [41]. The 3,4-DMCA in coffee extract is found as a conjugate with quinic acid, but after ingestion of coffee, all 3,4-DMCA is found in free-form in plasma samples [41]. It has been shown that hydrolysis of 3,4-DMCA derivatives by intestinal esterases occurs in the small intestine [48].

Finally, it is worth mentioning curcumin (diferuloylmethane), which is not formally a HCA derivative, but has common structural features with them (Figure 1). Curcumin is a natural dye and is derived from the roots of the *Curcuma longa* plant. A previous study has shown the ability of curcumin to overcome the blood–brain barrier, which indicates its potential to be used for the prevention and treatment of various neurodegenerative diseases [49]. In further sections, we discuss the potential application of curcumin and its comparison with HCA derivatives.

## 3. Synthesis and Properties of Natural and Synthetic Hydroxycinnamic Acid Derivatives 

**Figure 2:**
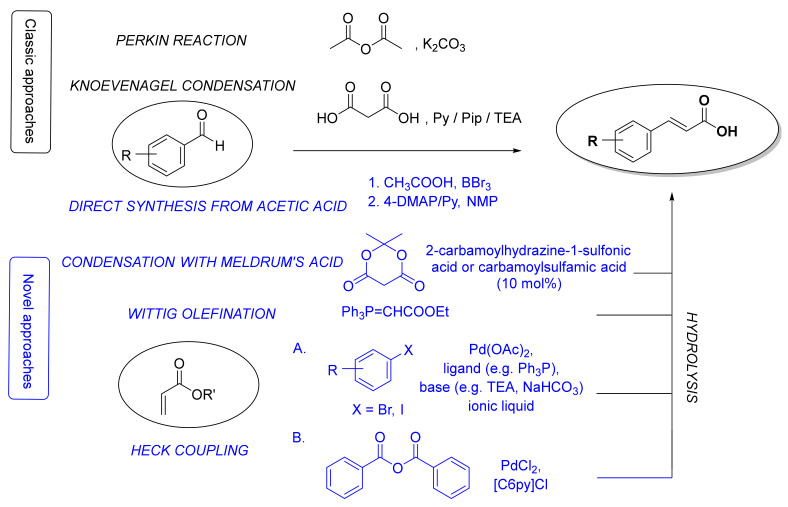
The most common classic (in black) and novel (in blue) synthetic methods for the synthesis of cinnamic acid moieties. Py, pyridine; Pip, piperidine; TEA, triethylamine; 4-DMAP, 4-dimethylaminopyridine; NMP, *N*-methyl-2-pyrrolidone; [C6Py]Cl, *N*-hexylpyridinium chloride.

There are three major routes for the preparation of the fundamental cinnamic acid scaffold: carboxyl group formation, synthesis of a propenoic acid fragment or addition of an aromatic radical on the propenoic acid moiety [50]. The most common and straightforward methods for the synthesis of cinnamic acid itself is via the Perkin reaction [51] or Knoevenagel condensation [52] starting from benzaldehyde (Figure 2). Even though the latter procedure can also be used for the synthesis of HCA derivatives, many other methods for constructing a cinnamoyl moiety have been also developed (Figure 2), e.g., reaction of benzaldehydes with Meldrum’s acid in the presence of catalytic amounts of 2-carbamoylhydrazine-1-sulfonic acid and carbamoylsulfamic acid [53], Wittig olefination reaction of benzaldehydes [54,55], Heck coupling of aryl halides or benzoic anhydride with alkenes [56], and straightforward synthesis from aromatic aldehydes, aliphatic carboxylic acids and boron tribromide using 4-dimethylaminopyridine and pyridine as bases [57]. Here, we will focus only on synthetic methods for the preparation of HCAs and their derivatives. 

**Figure 3:**
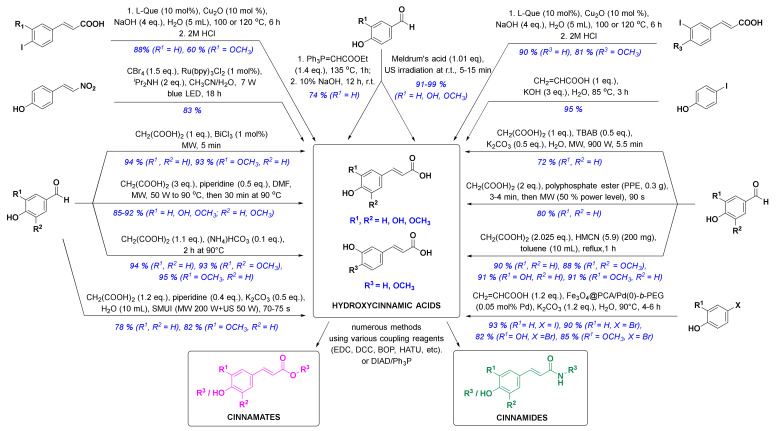
Novel synthetic methods for the synthesis of HCA derivatives (reagents are presented in black, yields and substituents in blue). l-Que, l-(−)-quebrachitol; LED, light emitting diode; MW, microwave irradiation; DMF, dimethylformamide; SMUI, simultaneous microwaves-ultrasound irradiation; US, ultrasound; TBAB, tetrabutylammonium bromide; PPE, polyphosphate ester; HMCN, hexagonal mesoporous carbon nitride; Fe_3_O_4_@PCA/Pd(0)-b-PEG, hyperbranched poly(ethylene glycol)-block-poly(citric acid)-functionalized Fe_3_O_4_ magnetic palladium nanoparticles; EDC, 1-(3-dimethylaminopropyl)-3-ethylcarbodiimide; DCC, dicyclohexylcarbodiimide; BOP, benzotriazol-1-yloxy)tris(dimethylamino)phosphonium hexafluorophosphate; HATU, 1-(bis(dimethylamino)methylene)-1*H*-[1,2,3]triazolo[4 ,5-*b*]pyridine-1-ium 3-oxide hexafluorophosphate; DIAD, diisopropyl azodicarboxylate.

In the past ten years, there has been a trend to replace the classic synthetic methodologies with novel and preferably eco-friendly/green procedures involving mild reaction conditions, minimal waste and low energy consumption. One successful eco-sustainable example is visible light photoredox catalysis (Figure 3), which implements visible light as a clean and cheap energy source, and CBr_4_ in alkaline aqueous solution providing the carboxylic acid moiety. (*E*)-4-(2-nitrovinyl)phenol and CBr_4_ as coupling partners were thus used for a highly stereoselective synthesis of (*E*)-*p*-coumaric acid in high 83% yield [58]. New green chemistry approaches have commonly employed water as the main solvent. Thiemann and coworkers described the Wittig reaction between 4-hydroxybenzaldehyde and ethoxymethylidenetriphenylphosphorane in 10% aqueous sodium hydroxide (Figure 3), which enabled one-pot olefination-hydrolysis sequence reactions leading to *p*-coumaric acid in situ in 74% yield [55]. The main methoxycinnamic acids were also synthesized, e.g., 3,4-dimethoxycinnamic acid (3,4-DCMA) was obtained in excellent 96% yield. Similarly, alkaline aqueous solution was used for the Heck coupling reaction between 4-iodophenol and acrylic acid, in which *p*-coumaric acid was prepared in excellent 95% yield in the presence of potassium hydroxide [59]. A green chemistry approach employing edible fruits and vegetable, liqueurs, and waste waters (buttermilk and residues of olive processing) as solvents with Meldrum’s acid and appropriate aldehyde under ultrasound irradiation provided *p*-coumaric, caffeic and ferulic acids in excellent yields [60]. Another modern strategy was to perform the reactions under solvent-free conditions. One such example was developed by van Schijndel and coauthors using environmentally benign amines or ammonium salts which afforded α,β-unsaturated acids from corresponding benzaldehydes and malonic acid via Knoevenagel condensation. Sinapic, ferulic and *p*-coumaric acid were prepared in moderate to high yields from 3,5-dimethoxy-4-hydroxy-, 3-methoxy-4-hydroxy- and 4-hydroxy-benzaldehydes, respectively; however, with higher temperatures (i.e., 140 °C), second decarboxylation usually occurred [61]. Recently, van Schijndel and coauthors published a similar procedure at 90 °C that led to aforementioned HCAs in excellent yields (Figure 3) [62]. Furthermore, to limit the decarboxylation of the resulting HCAs, another approach employing microwave irradiation was presented in the literature. Microwave-assisted Knoevenagel–Doebner condensation of various *par*a-hydroxybenzaldehydes with malonic acid and piperidine as a base in dimethylformamide (Figure 3) afforded *p*-coumaric, caffeic, ferulic and sinapic acids in 92, 85, 89, and 90% yield, respectively [63]. Furthermore, the addition of tetrabutylammonium bromide (TBAB) in Knoevenagel reaction afforded quick, economic and eco-friendly microwave-assisted synthesis of *p*-coumaric acid from 4-hydroxybenzaldehyde and malonic acid in 72% yield using potassium carbonate as a base and distilled water as a solvent [64]. *p*-Coumaric, 4-methoxycinnamic and 3,4-dimethoxycinnamic acids were also synthesized in 80, 85 and 92% yield in solvent-free conditions and microwave irradiation using polyphosphate ester as a reaction mediator and catalyst [65]. Recent studies on HCA synthesis have also focused on the development of novel catalysts for Knoevenagel–Doebner condensation or the Heck reaction (Figure 3), e.g., nanochannels of hexagonal mesoporous carbon nitride with optimized pore sizes [66,67], amphiphilic and hyperbranched polymer-functionalized magnetic nanoparticles with palladium [68], and bismuth(III) chloride [69]. Using these catalysts, all major HCAs and methoxycinnamic acids were synthesized (Figure 3). The reaction of aryl iodides in NaOH (aq) with copper as a catalyst and l-(−)-quebrachitol as a ligand (Figure 3) afforded ferulic, isoferulic and 3- and 4-coumaric acids in high yields [70]. Another interesting approach was combining microwave and ultrasound irradiation to accelerate the Knoevenagel–Doebner reaction in aqueous media [71]. The optimized green method provides the rapid synthesis of HCAs with low energy consumption, minimal waste and without any organic solvents and expensive catalysts. One recent synthetic methodology also employed a recyclable silicon-containing biphenyl-based template affording the Pd-catalyzed *para* C-H functionalization of numerous phenolic compounds as starting materials (e.g. phenol and 2-methoxyphenol were converted to *p-*coumaric acid and ferulic acid in both 4 steps and 47% yields, respectively) [72]. And last but not least, HCAs and derivatives can also be prepared via enzymatic reactions [73,74,75], most commonly performed by microbes, e.g., bacteria.

Natural HCAs possess numerous biological activities that have been extensively reviewed in several publications [76,77,78,79,80,81,82,83,84]. In the last 20 years, the chemistry-driven derivatization of HCAs has led to a large number of semi-synthetic and synthetic HCA derivatives. In order to obtain specific biological effects, HCAs were most commonly converted to corresponding esters or amides. For example, an ester of *p*-coumaric acid (methyl (*E*)-(3-(4-hydroxyphenyl)acryloyl)-l-phenylalaninate) and two esters of caffeic acid (dibenzyl (*E*)-(3-(3,4-dihydroxyphenyl)acryloyl)-l-aspartate and methyl (*E*)-(3-(3,4-dihydroxyphenyl)acryloyl)-l-alaninat) were synthesized by condensation of the corresponding cinnamic acids with amino acid esters using 1-(3-dimethylaminopropyl)-3-ethylcarbodiimide hydrochloride (EDC), 1-hydroxybenzotriazole (HOBT), and triethylamine in methylene chloride [85]. The first two HCA esters exhibited anti-atherosclerotic activity via inhibitory effects on acyl-CoA:cholesterol acyltransferase involved in cellular cholesterol storage and transport, and inhibition of LDL-oxidation and high-density lipoprotein particle size rearrangement [85]. The same coupling method (for the synthesis of amides) or alternative esterification utilizing diisopropyl azodicarboxylate and triphenylphosphine was used for preparation of a series of caffeic, ferulic and *p*-coumaric acids amides with biogenic amines (serotonin, dopamine, tyramine, vanillylamine) or esters with 3,4-dihydroxyphenethyl, 4-hydroxyphenethyl, and phenethyl alcohols [86]. Compounds containing catechol, *o*-methoxyphenol or 5-hydroxyindole moieties exhibited potent 1,1-diphenyl-2-picrylhydrazyl free radical scavenging activity (amides more potent than esters). Some HCA derivatives also showed potent and selective MAO-B (phenethyl (*E*)-3-(4-hydroxyphenyl)acrylate being the most potent one) or moderate BChE (e.g., 3,4-dihydroxyphenethyl (*E*)-3-(4-hydroxyphenyl)acrylate) inhibitory activity [86]. The synthesis of phenethyl *E*-3-(4-hydroxy-3-methoxyphenyl)acrylate and *E*-3-(4-hydroxy-3-methoxyphenyl)-*N*-phenethylacrylamide from ferulic acid with phenethyl alcohol and phenethylamine, respectively, was performed via indirect reaction, including acetylation for hydroxyl group protection, acid chloride formation, esterification/amidation, and deacetylation as a deprotection reaction [87]. Both compounds showed anticancer activity against P388 murine leukemia cells. Other coupling reagents, i.e., benzotriazol-1-yloxy)tris(dimethylamino)phosphonium hexafluorophosphate(BOP), 1-(bis(dimethylamino)methylene)-1*H*-[1,2,3]triazolo[4,5-*b*]pyridine-1-ium 3-oxide hexafluorophosphate (HATU), and dicyclohexylcarbodiimide (DCC) have been used for the preparation of ferulic [88] and caffeic acid esters and amides [89]. The nucleophilic hydroxyl groups were often protected via acetylation by acetic anhydride in the presence of pyridine [87,90,91] or other bases [92,93]. Other methods for the preparation of HCA esters and amides have been used and were thoroughly reviewed by de Armas-Ricard and co-authors in 2019 [89].

As previously described, esters and amides of coumaric, ferulic and caffeic acid possess numerous biological activities, e.g., antioxidant [82,83,86,89,94,95,96], anticancer [78,87,92], antibacterial [82,97,98,99], antifungal [100,101,102], antiviral [82,90,103], anti-inflammatory [104,105,106], and many other activities [84,107,108]. The most commonly described health beneficial properties of HCAs and their derivatives is antioxidant activity due to the phenolic hydroxyl group(s) on the main aromatic ring, which possess(es) the ability to react with free radicals and reactive oxygen species forming a resonance-stabilized phenoxyl radical. Furthermore, propenoic side chain involvement in the stabilization of a phenoxyl radical via a conjugated double bond also contributes to antioxidant properties of HCAs [82,109]. Their antioxidant potential is affected by substituents on the phenyl ring (e.g., methoxy group on position 3 increases the antioxidant potency due to electron-donating properties).

The substitution on the aromatic ring has also a high impact on their physico-chemical properties. Among the main cinnamic acid derivatives, higher solubility was observed for caffeic acid, followed by ferulic and *trans*-cinnamic acids due to the extent of hydrogen bonding with water—a higher number of hydroxyl groups leads to increased association interactions with water [110]. The hydroxy substituent also shows a significant impact on the acidity of HCAs [111]. For coumaric acids, the pKa values for carboxylic acid group (pKa_1_) increases in the following order: *orto* < *meta* < *para* (4.11, 4.49 and 4.70, respectively), whereas for phenolic group (pKa_2_) the *m*-coumaric acid is the least acidic (pKa_2_ = 10.39) [111]. On the other hand, the apparent acid dissociation constants (pKa_app_) of *trans*-cinnamic, ferulic and caffeic acids were determined as 4.55, 4.61 and 4.77, respectively [110].

## 4. Molecular Modeling of the Interaction of Hydroxycinnamic Acid Derivatives and Various Forms of Amyloidogenic Proteins

Molecular modeling of small molecule interactions with target proteins is currently a method used first, and sometimes as a stand-alone, for the evaluation of new drug candidates. This approach is hard to apply in the case of many amyloidogenic proteins since they are either completely or partially naturally unfolded. The inability to crystallize proteins with intrinsically unfolded structure means there is no data available on their tertiary structure based on X-ray crystallography analysis. Nevertheless, a few studies have investigated the molecular modeling using specific structured parts of amyloidogenic proteins present either in monomers, oligomers, or fibrillar aggregates. Herein we will review works concerning molecular modeling of the interaction of HCA derivatives and similar compounds with PrP and α-synuclein, the proteins that are the main focus of our research.

### 4.1. Molecular Modeling of the Interaction of Hydroxycinnamic Acid Derivatives and Prion Protein Monomers

**Figure 4:**
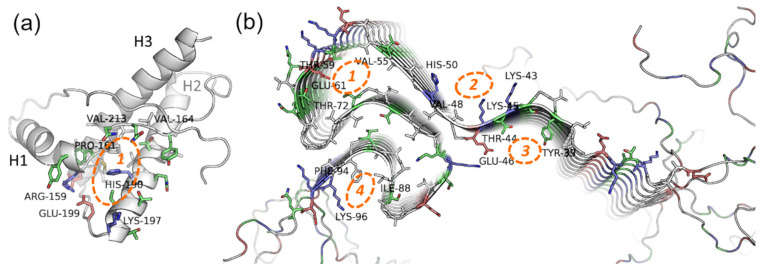
Potential binding sites on the (**a**) native prion protein and (**b**) fibrils of α-synuclein. PDB IDs 1tqb (ovine PrP) and 2n0a (fibrils of human α-synuclein). Blue, red, green, and grey represent basic, acidic, polar, and hydrophobic residues respectively. For the details about the “hotspot” region of PrP (1), see the details in the text; the binding sites localization on α-synuclein fibrils is based on data from [117,118].

PrP is only partially intrinsically unfolded. Although PrP has an unstructured region, its main physiological conformation (so-called PrP^C^, cellular form) has an α-helical structure. In certain conditions, i.e., in the presence of PrP in amyloid conformation or some metal ions, PrP^C^ can be rich in β-structures (PrP^Sc^, scrapie form), with a propensity for amyloid aggregation [112,113]. The structure of the PrP ordered fragment (a.a.r. 134–231 in human PrP) is known [114], hence modeling approaches (first and foremost docking and virtual screening) seem like prospective methods in the search for potential anti-prion compounds. The main binding site for these compounds is a cavity in-between the interfaces of the H1 α-helix and S2 β-strand, and H2 and H3 α-helices (Figure 4a)—the so-called “hotspot” region of the protein that is proposed to play a key role in the amyloidogenic conversion of PrP [115,116].

The majority of anti-prion compounds, including polyphenols, bind precisely to this partially hydrophobic cavity surrounded by polar and charged residues. For example, according to docking and in some cases experimental confirmation, it is the binding site of curcumin and other HCA derivatives [119,120], chalcone derivatives [121,122], and other polyphenolic compounds. This was additionally confirmed by the effectiveness of the anti-prion activity of the compounds selected in this work by virtual screening. A differing binding site was proposed for humic acids, which are polyanions with many phenyl groups (including HCA derivatives) [123]. Such a change is probably due to its anionic nature and affinity to the positively-charged parts of the PrP surface. Notably, although binding sites of many anti-amyloid compounds are similar, the mechanism of action may differ. For instance, in the paper by Zhou et al. [124] based on molecular dynamic simulations data on a few well-known potential inhibitors of amyloidogenic aggregation of PrP, it was proposed that 1′,4′,5′,1,4,5-hexamethoxychalcone stabilizes the hydrophobic core of PrP, while another promising compound GN8 stabilizes the hydrophobic core, as well as the flexible C-terminal end of the H2 helix. Nevertheless, in most cases, research is limited to docking and virtual screening of the compounds; hence, the detailed mechanism of action of the potential anti-prion compounds (and, in particular, HCA derivatives) has not been elucidated. In the future, this research should conduct molecular modeling of a broad spectrum of synthetic and naturally occurring HCA derivatives’ interactions with the structured domain of PrP and, possibly, with specific elements of its amyloid structures. This modeling approach will uncover the role of particular groups of HCA derivatives in the anti-amyloid effect and select the most prospective compounds.

### 4.2. Molecular Modeling of the Interaction of Hydroxycinnamic Acid Derivatives and Different Forms of α-Synuclein

Molecular modeling of HCA derivatives with α-synuclein is hindered by the fact that, unlike PrP, it is a completely naturally unfolded protein in physiological conformation. It leads to the complete absence of docking or other modeling works on the binding of HCA derivatives with native monomeric α-synuclein, and, as a consequence, a lack of attempts to search for potential anti-aggregation compounds via virtual screening. Nevertheless, much experimental data points to anti-aggregation activity of polyphenolic compounds on α-synuclein, including curcumin, rosmarinic acid, and other HCA derivatives [21,125,126,127,128]. However, the detailed mechanism of this activity is unknown. Different papers suggest the influence of various polyphenols on various stages of amyloidogenic transformation of α-synuclein (such as inhibition of oligomerization, or, in contrast, conversion of oligomers to fibrils with an overall decrease in cytotoxicity). Singh et al. [21] demonstrated that curcumin binding is stronger as a result of α-synuclein oligomerization, but no mechanism of interaction was proposed. We will further discuss this experimental work in Section 6.

A number of papers have proposed using molecular modeling to search for the binding site of potential anti-aggregation compounds via docking to the structure of micelle-bound human α-synuclein (PDB ID 1xq8):caffeic acid [129], curcumin, and other polyphenolic compounds [130,131]. It is worth noting that this structure is reflective only of one of the α-synuclein forms and does not reproduce its behavior in the prone-to-aggregation state. This makes docking pointless for the search of potential anti-amyloid compounds, especially in cases of docking to the unstructured C-terminal domain. The utilization of ensemble docking looks more promising when potential ligands are docked not to the one “initial” structure, but to the ensemble of conformations gathered by molecular dynamic simulations [132]. This approach was used, for example, to predict the binding site of phenolic compounds (noradrenaline and scutellarin) [133,134]. Difficulties of this approach include the complexity of the method and the fact that the quality of the result depends on how close to reality the ensemble of conformations is.

Finally, docking of potential ligands can be done to the structure of the already formed α-synuclein fibrils, which have a few potential binding sites for small molecules in the β-structured NAC-domain and unstructured tails of the polypeptide chain [117] (Figure 4b). This approach seems appropriate for the compounds that are expected to bind not to monomeric, but oligomeric forms or formed fibrils of α-synuclein, and potentially influence their assembly rate or disintegrate them. For instance, in our work [118], a few HCA and caffeic acid derivatives (ferulic acid, 3-methoxy-4-acetamidoxycinnamic acid, 3,4-DMCA) were shown to not bind to native monomeric α-synuclein, although they exerted a pronounced anti-amyloid effect. Based on docking to fibrils the hypothesis was formulated that these compounds bind to prefibrillar oligomers or small fibrils, influencing further aggregation. Similar to PrP, it seems worthwhile to widen the spectrum of HCA derivatives used for molecular modeling with the objective to uncover precise mechanisms of their binding to amyloid forms of α-synuclein.

## 5. Hydroxycinnamic Acid Derivatives Modulating the Pathological Transformation of Prion Protein

PrP is an amyloidogenic protein whose amyloid conversion ultimately leads to the development of a number of neurodegenerative diseases of animals (mad cow disease) and humans (Creutzfeldt–Jakob disease, fatal familial insomnia, etc.) [2,112]. Although there are many approaches to reduce the toxic effects of prions, this problem has not yet been solved. The simplest method for the prevention and treatment of prion diseases could be the use of small molecules that selectively interact with certain forms of PrP and prevent its amyloidization. Particularly promising are natural compounds or their natural metabolites in the body, which include HCA derivatives.

**Figure 5:**
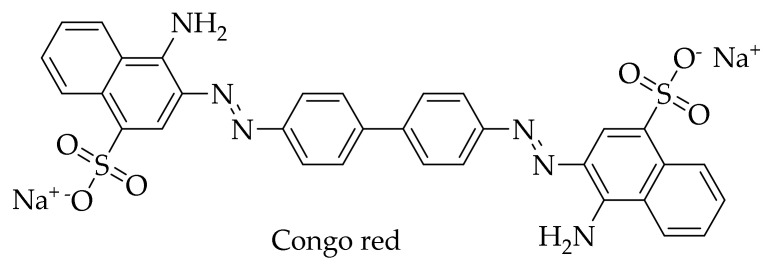
Structure of Congo red dye.

Interest in HCA derivatives arose after the work on the anti-amyloid action of the dye Congo red (Figure 5). Since 1922, Congo red staining has been used to detect protein aggregates with amyloid structure in tissues [135]. At the end of the last century, scientists found that this dye is also able to block the formation of an abnormal form of PrP (PrP^Sc^), which is resistant to proteinase K, in neuroblastoma cells infected by mouse prion, and even free these cells of prion infection [136]. Unfortunately, subsequent experiments showed that Congo red is toxic to mammals, which makes it impossible to use for the treatment of prion diseases [137,138]. Since it is considered that the location of the central phenyl rings in the Congo red molecule and their mobility are important for inhibition of the formation of PrP^Sc^ [139], scientists drew attention to the dye-related compounds with a similar structure. First of all, the anti-amyloid activity of curcumin was studied, a compound with known diverse biological activities. Curcumin (Figure 1) resembles Congo red (Figure 5) in its structure—both molecules consist of benzene rings joined by mobile linkers. In addition, curcumin has no apparent toxicity and is widely used as a spice [14,140].

Curcumin, similarly to Congo red, was shown to inhibit the accumulation of the dangerous form of PrP^Sc^ in prion-infected neuroblastoma cells (IC_50_ 10 nM) [141], and compete with the dye in binding to the β-form of oligomeric and fibrillar PrP. Curcumin also interacts with the α-helical intermediate of PrP [142], which is formed at acidic pH, and which may hinder its further interaction with a “foci” of amyloid aggregation [19].

A study conducted in 2013 in a cell-free system on a mouse PrP showed that 20 μM curcumin is able to reduce the formation of amyloid fibrils. In addition, PrP aggregates obtained in the presence of curcumin were not susceptible to proteinase K, in contrast to the true amyloid forms of the protein that are known to be resistant. Moreover, 2.5 μM curcumin rescued mouse neuroblastoma (N2a) cells from apoptosis caused by the accumulation of amyloid structures [20].

On the contrary, the results of in vivo studies have been contradictory. Caughey et al. found that a diet with unlimited food containing a 2% mass fraction of curcumin did not significantly affect the development of prion infection in hamsters [141]. On the other hand, Riemer et al. demonstrated that mice infected with prion and given a low dose of curcumin (50 mg per kg of body weight) lived 12 days longer than control mice (*p* < 0.01) [143].

These curcumin deficiencies stimulated the search for compounds of similar structure, but with better water solubility and bioavailability. Given the “cumbersome” nature of the curcumin molecule and its symmetrical structure, it has been suggested that some HCA derivatives, which actually represent half the curcumin molecule, may also have an anti-amyloid effect. First of all, 3,4-DMCA was docked; 3,4-DMCA also has much better solubility in water—the apparent solubility of 3,4-DMCA is 5 mM against that of 0.5 mM for curcumin. Although the binding of 3,4-DMCA to the site found for curcumin cannot be excluded, a more energy-efficient site between H2 and H3 helices was found when modeling the binding of 3,4-DMCA to PrP, and the suggested binding mode is supported by hydrogen bonds and electrostatic interactions with R139 and N162 residues [119] with dG values of −4.5 kcal/mol against −3 kcal/mol for curcumin (Figure 4).

Experimental studies confirmed the results of molecular modeling of the interaction of 3,4-DMCA with a prion monomer. Using various approaches, the effects of 3,4-DMCA, ferulic acid, and 7 chemical derivatives bearing different substituents in *o*-, *m*-, and *p*- positions of cinnamic acid were studied for anti-prion activity [119,144]. Among naturally occurring HCAs, the most promising effect was shown for 3,4-DMCA. It was found that 3,4-DMCA binds efficiently to a PrP with a dissociation constant of 405 nM. Using isothermal titration calorimetry, dynamic light scattering, the thioflavin T (ThT) assay, and circular dichroism spectroscopy, it was shown that HCAs are able to partially suppress the oligomerization and fibrillation of PrP induced by various factors. In particular, it was shown that they suppress the pathological transformation of PrP under the closest to natural conditions—when preformed amyloid fibrils are added to PrP monomers, i.e., during so-called seeding. The studied derivatives were also able to increase the viability of SH-SY5Y neuroblastoma cells after the addition of prion oligomers, which indicates a decrease in prion neurotoxicity under the influence of some HCA derivatives [119,144].

HCA derivatives can have another effect on the progress of neurodegenerative diseases, in addition to direct interaction with prion molecules and their aggregates. For example, it has been shown that caffeic acid can increase neuronal viability due to the inhibition of apoptosis induced by the PrP106-126 peptide [145]. In this case, caffeic acid serves as a specific inhibitor of 5-lipoxygenase (5-LOX), through which apoptosis is triggered.

The development of drugs for prion diseases is fraught with a number of obstacles, such as the low incidence of spontaneous forms of prion pathologies in humans (in the absence of infectious outbreaks), the very long latent period before the onset of clinical symptoms and the incurability at clinically obvious stages due to practically irreversible molecular and cellular disorders. Therefore, the possibility of safe, regular and prolonged use, as in the case of 3,4-DMCA and ferulic acids from coffee and other dietary plants, looks promising for the prevention and inhibition of the rate of development of diseases at undetectable stages.

## 6. Hydroxycinnamic Acid Derivatives Modulating the Pathological Transformation of α-Synuclein

α-synuclein is the main amyloidogenic protein implicated in the pathogenesis of Parkinson’s disease and other synucleinopathies. Parkinson’s disease is characterized by the formation of protein aggregates called Lewy bodies, mainly consisting of α-synuclein protein, in the nerve cells. For a long time, the accumulation of α-synuclein fibrils was believed to be the main cause of the pathological changes associated with this neurodegenerative disease. However, in recent years it has been shown that the toxicity of α-synuclein is the result of the formation of oligomeric intermediates formed in the process of amyloid aggregation [146]. α-synuclein fibrillation intermediates (oligomers, protofibrils) and final mature fibrils display different toxicities [147]. At the same time, the deposition of α-synuclein fibrils in Lewy bodies can be regarded as a protective mechanism for reducing the toxicity of oligomeric forms [148,149]. Thus, it is now generally accepted that the formation of amyloid α-synuclein oligomers is crucial in synucleinopathies, including Parkinson’s disease [150,151].

Numerous epidemiological and experimental studies indicate that daily consumption of polyphenolic compounds with food protects against neurodegeneration [152,153,154]. Polyphenols are also known to possess antioxidant, anti-inflammatory and chelating activities, which make it possible to regard these compounds as potential agents for the prevention and treatment of neurodegenerative diseases, including Parkinson’s disease [155,156]. In early studies on the mechanisms of action of polyphenolic compounds, it was generally assumed that their action was due to their antioxidant activity. This explained the effect of curcumin described in the previous sections, as well as the polyphenol baicalein contained in the root of *S. baicalensis* (*Scutellaria radix*). Baicalein is actively used in traditional Chinese medicine due to its antioxidant, anti-inflammatory, and anticarcinogenic properties [157,158]. However, information about the direct effect of polyphenolic compounds on the pathological transformation of amyloidogenic proteins has been gradually accumulating. Thus, many phenolic and polyphenolic compounds were proven to inhibit the formation of amyloid protofilaments and α-synuclein fibrils by stabilizing monomers or remodeling and inactivating toxic protein oligomers [159].

Masuda et al. [160] tested 79 ligands from various chemical classes of compounds (including polyphenols, benzothiazoles, terpenoids, steroids, porphyrins, lignans, phenothiazines, polyene macrolides, and Congo red and its derivatives) on their ability to inhibit α-synuclein fibrillation. Of the 39 polyphenol compounds tested, 26 were found to inhibit α-synuclein aggregation. These studies have shown that it is very likely we will find potential inhibitors of α-synuclein amyloid aggregation among polyphenols.

The combination of nuclear magnetic resonance, circular dichroism spectroscopy, and electron microscopy methods has demonstrated that in the presence of polyphenolic compounds, the assembly of α-synuclein oligomers and fibrils is destabilized due to the presence of aromatic and hydroxyl groups on the phenyl ring of the ligands [161]. This is supported by the notion that the number of hydroxyl groups influences the potency of fibrillation inhibition by tea polyphenols [162]. Presumably, the aromatic rings of polyphenols can interact with the monomeric and oligomeric forms of α-synuclein sterically inhibiting further protein aggregation [163].

Many authors especially emphasize the polyphenols baicalein and curcumin as inhibitors of α-synuclein fibrillation. For baicalein, the therapeutic effects in Alzheimer’s and Parkinson’s diseases [164] and the ability to inhibit aggregation of both PrP [165] and α-synuclein [166] have been demonstrated. This compound inhibits amyloid aggregation of α-synuclein in vitro [167] and in vivo in mouse and rat models [166,168]. During in vitro inhibition of α-synuclein aggregation by baicalein, strong binding of polyphenol to the unstructured C-terminus of the protein [160] and stabilization of oligomers preventing further fibril formation [169] were assumed. The binding of the compound to the C-terminus of α-synuclein was also confirmed by the experiments of Meng et al. [170], showing in vitro the lack of binding of baicalein to the mutant forms 1–103 and 1–122 of the C-terminally truncated α-synuclein. The authors also note that the strong inhibitory effect of the compound is the result of the formation of a Schiff base between amino groups of the protein and baicalein quinones [170].

The anti-amyloid effect of curcumin is associated with an increase in the solubility of α-synuclein monomers dose-dependently inhibiting protein oligomerization. According to published data curcumin binds to the α-synuclein monomer, with a dissociation constant of 10^−5^ M, affecting the configuration of the molecule and thus blocking oligomerization and fibrillation [171]. Using in vitro experiments on various mutant forms of α-synuclein, it has been shown that the binding of curcumin to the protein occurs in a hydrophobic non-amyloid-β component region 60–100, in which 15 aliphatic amino acids, tryptophan in position 94 or 3 alanines in position 89–91 may serve as possible binding sites [171], which also correlates with the results of molecular docking for HCA derivatives carried out using fibril structures [118].

The effect of HCA derivatives, the structure of which is similar to a half of the curcumin molecule, and structurally similar compounds on the amyloid transformation of α-synuclein, was studied by several groups [127,128,155,172,173,174,175,176,177] and is summarized in Table 1.

The most active in inhibiting α-synuclein fibrillation was ferulic acid with a half-maximal inhibitory concentration of 0.8–13 µM reported in different studies [118,161]. It is noteworthy that some of the reported compounds such as gallic and caffeic acids were most effective in preventing seeding of aggregation by preformed fibrils, then in inhibiting aggregation when added to the monomeric α-synuclein [127]. Many of the aforementioned compounds were also active in disintegration of fibrils, including ferulic, gallic, protocatechuic acids, and hydroxytyrosol. Moreover, protocatechuic acid was more effective in fibril disintegration than in preventing amyloid aggregation [173].

Data on the interaction with monomers and inhibition of α-synuclein monomers is less comprehensive. In one study, no interaction of gallic acid with α-synuclein monomers was shown [173], while other researchers proposed that transient interactions with N- and C-terminal domains and gallic acid were responsible for averting α-synuclein transformation into a partially folded intermediate, necessary for further fibrillation [172]. The first group has also shown the formation of soluble non-toxic oligomers with no β-structure, which is stable in denaturing conditions [173]. Higher concentrations of gallic acid led to the inhibition of oligomerization, while lower concentrations promoted and stabilized oligomers [173]. Addition of 3,4-dihydroxyphenylacetic acid led to the formation of oligomers that dissipated into monomeric α-synuclein upon dilution and separation by size exclusion chromatography [175], and the compound itself bound to the N-terminus of α-synuclein. Hindrance of oligomerization was also demonstrated for ferulic acid [128], where the diameter and height of the aggregates decreased according to electron and atomic force microscopy. Rosmarinic acid, a caffeic acid ester of 3-(3,4-dihydroxyphenyl)lactic acid, did not bind to monomeric α-synuclein [128], although previous data indicated that it interacts with the N-terminus [178]. To sum up, the mechanism of α-synuclein fibrillation inhibition by HCA derivatives and structurally similar compounds remains unclear. On one hand, data points to binding to the monomer and stabilization of non-toxic oligomers, while on the other hand, there are results in favor of no binding to the monomer and more active disintegration of fibrils and prevention of seeding, rather than prevention of fibrillation from the monomeric protein.

A comparative analysis of the effect of 9 HCA and caffeic acid derivatives on the ability to prevent α-synuclein amyloid transformation was performed by our group: two naturally occurring (ferulic acid and 3,4-DMCA) and seven synthesized compounds [118]. Synthetic compounds were investigated in order to find out which substitutions in the structure are important for the implementation of the studied effects. It was shown that two natural compounds, as well as synthetic 3-methoxy-4-acetamidoxycinnamic acid, are capable of dose-dependently inhibiting α-synuclein fibrillation detected by ThT fluorescence. The ability to inhibit α-synuclein aggregation decreases in the following order: ferulic ≈ 3-methoxy-4-acetamidoxycinnamic >> 3,4-DMCA, with half maximal inhibitory concentrations of 13 ± 2 μM, 50 ± 2 μM and 251 ± 41 μM accordingly. The remaining six compounds have almost no effect on aggregation. A-synuclein fibrils grown in the presence of three active HCA derivatives have less resistance to proteolysis by proteinase K, altered circular dichroism spectra and smaller sizes according to scanning ion-conducting microscopy. It is important to highlight that even when using millimolar concentrations of the three HCA derivatives (1.4 mM), they do not possess a cytotoxic effect towards the SH-SY5Y cell line. It should be noted that we detected no binding of these compounds to monomeric forms of α-synuclein, which indicates their interaction with β-rich forms in accordance with the results of molecular modeling (described in detail in Section 4).

Considering the possible mechanism of action of HCA derivatives and structurally similar compounds, a correlation is noted between the number of hydroxyl groups in the compounds and their efficiency in inhibiting α-synuclein aggregation: the more groups, the stronger the effect, while identical molecules with a hydroxyl group replaced by a methyl group no longer inhibit fibrillation [179]. Given the importance of the inhibitors’ -OH groups, it is believed that binding to the aromatic residues of α-synuclein via π–π interactions plays a key role [173]. The interaction of the potential fibrillation inhibitors through aromatic rings with aliphatic amino acids forms a hydrophobic cluster, which leads to the inaccessibility of the α-synuclein hydrophobic region for the interaction with other monomers [171]. Having tested 14 compounds as potential inhibitors of α-synuclein amyloid aggregation, Caruana et al. deduced two general conditions for the most powerful inhibitors: (1) aromatic recognition elements that would allow non-covalent binding to the α-synuclein monomer/oligomer, and (2) hydroxyl groups on a single phenyl ring that would prevent the self-assembly process and/or destabilize its structure [159,163]. These assumptions were confirmed by our results screening the anti-amyloid action of two naturally occurring and seven synthetic HCA derivatives. They were also confirmed by other data: less potent in inhibiting α-synuclein aggregation, O-methyldehydrozingerone and 3,4-DMCA have methoxy groups instead of hydroxyl attached to the phenolic ring; 3-hydroxybenzoic and 2-hydroxybenzoic (salicylic) acids with only one hydroxyl group were not able to inhibit aggregation [173]. Another assumption was made by considering positions of multiple hydroxyl groups on the ring. Generally, compounds with neighboring –OH groups were more effective [173]. Analyzing the activity of different compounds, attention is brought to another correlation: the extent of activity correlates with the presence of an aliphatic side chain on the phenolic ring. For example, less active then caffeic acid, protocatehuic acid differs from it only in the length of the aliphatic side chain and the presence of a double bond in it. Similarly, 2,5-dihydroxybenzoic acid, which inhibits aggregation by only 30% [173], has a shorter side chain than the more effective homogentisic acid [175] with identical –OH group positions. Moreover, 3,4-dihydroxyphenylacetic acid is more effective than 3,4-dihydroxybenzoic [173,175]. These assumptions are pulled from the analysis of multiple papers; hence a comparative experimental study is needed to prove this concept.

As noted above, many HCA derivatives are found in black and especially green coffee beans [42,43,44], and their concentration in the blood increases significantly when drinking coffee [45,46]. It has been shown that with daily coffee intake of 1 g/L in the blood plasma of mice injected with α-synuclein fibrils 7 days after the start of coffee treatment, the concentration of caffeine and coffee metabolites was 0.4–2 mg/L and the amount of pathological aggregates of α-synuclein A53T, prone to aggregation mutant, decreased [180]. Caffeine has also been shown to reduce the risk of developing Parkinson’s disease. Moreover, during mitochondrial stress, a prominent feature of synucleinopathies [181], caffeine acts as an antioxidant [182]. In experiments with recombinant α-synuclein in yeast and mouse models, it was found that caffeine has a double effect: on the one hand, it accelerated the aggregation process, but on the other hand, caffeine changed the shape of mature aggregates. Aggregates formed in the presence of caffeine are characterized by amorphous morphology [183].

The concentration of caffeine in the blood of volunteers who took coffee thrice a day (160 mg of caffeine per serving) reached 4 μg/mL 100 min after coffee consumption [184]. In one experiment, polyphenol-rich foods were excluded from the volunteers’ diets, but a mixture of seven spices was added: turmeric, coriander seeds, caraway seeds, dried Indian gooseberries, cayenne pepper, cinnamon and cloves in a ratio of 8:4:4:4:2:1:1 with a total mass of 12 g. The concentration of cinnamic acid in blood plasma then reached 342 ± 83 nM 0.5–4 h after ingestion [185]. However, HCA derivatives, which prevent the pathological transformation of PrP [119] (see also Section 5) and α-synuclein [118], may be equally important coffee components. According to our preliminary data, synthetic 3-methoxy-4-acetamidoxycinnamic acid, which has pronounced anti-amyloid activity against α-synuclein, is present in coffee extracts. Given the natural origin of ferulic acid and 3,4-DMCA and their presence in human blood plasma [41], the selected compounds are promising agents for preventing and treating synucleinopathies.

## 7. Conclusions

The evidence reviewed above, including our findings, supports the notion that HCA derivatives are promising drug candidates for preventive and therapeutic use in neurodegenerative diseases of amyloidogenic nature. Although the main corpus of data on the application of HCA derivatives concerns their use in Parkinson’s and prion diseases, there are reasons to believe that they will be quite effective in other neurodegenerative pathologies. For example, a few studies have been published on anti-amyloid activity of HCA derivatives towards amyloid-β aggregates [186,187] implicated in Alzheimer’s disease development. In particular, ferulic acid can inhibit the formation of amyloid-β oligomers, while accelerating fibril formation [187]. There is also supporting data on ferulic acid shifting amyloid-β aggregation to an amorphous aggregation process [186]. Structural features of huntingtin, namely an extended polyglutamine repeat, does not allow prediction of the anti-aggregation influence of HCA derivatives on this protein, but this possibility should not be excluded before experimental investigation.

Notably, despite a pronounced anti-amyloid effect of HCA derivatives on PrP and α-synuclein, there are significant differences in the mechanisms of interaction of these ligands with the two proteins. Probably, HCA derivatives do not bind to completely disordered polypeptide chains but interact with structured elements. Such structured regions are present in PrP but are absent in α-synuclein monomers. For these reasons, HCA derivatives bind to monomeric, oligomeric, and fibrillar forms of PrP, preventing the formation of amyloid structures and disrupting already formed aggregates. At the same time, HCA derivatives interact only with the preformed amyloid structures of oligomers and fibrils of α-synuclein, preventing their further pathological transformation, but not with unstructured α-synuclein monomers. Structural features of the binding sites present in monomeric PrP or amyloid aggregates of α-synuclein also lead to a varying effectiveness of HCA derivatives on the formation of amyloid structures by these proteins. Interestingly, ferulic acid was not active in preventing PrP fibrillation [188], while successfully inhibiting α-synuclein amyloid aggregation [161]. Distinctive features of the action of HCA derivatives on various amyloidogenic proteins indicate the need for an expanded search for ligands with an optimal structure both among natural and synthesized compounds. It seems promising to obtain a wide range of synthetic analogues of HCA derivatives with the targeted replacement of individual moieties both to elucidate the mechanisms of their anti-amyloid action on different target proteins, and to select compounds with optimal properties.

However, the most promising compounds for the development of prophylaxis and treatment of amyloid neurodegenerative diseases are natural HCA derivatives. The advantage of natural HCA derivatives, contained in various foods and spices, is the centuries-old indication of their safety for the human body, as well as accumulated information about their ability to prevent or slow down a number of pathological processes. Moreover, there are pathways for the metabolism of certain HCA derivatives, which prevents their excessive accumulation in organs and tissues and, therefore, prevents overdose when taking such compounds as dietary supplements and medicines. It is also possible that a careful analysis of the content of HCA derivatives in various food products will reveal new compounds of this class. For instance, we have obtained preliminary data that synthetic 3-methoxy-4-acetamidoxycinnamic acid, which has a pronounced anti-amyloid effect against α-synuclein, is present in coffee extracts. It is possible that for some other synthetic HCA derivatives, there are naturally occurring analogs, which will facilitate the development of the drugs based on them.

Thus, further development of studies on HCA derivatives as anti-amyloid drugs can occur in the following areas:

1. Synthesis of a variety of HCA derivatives to elucidate the fundamental aspects of the mechanisms of their influence on the pathological transformation of amyloidogenic proteins.
2. Search for natural analogs of synthetic HCA derivatives in food products with a view to their further use for drug development.
3. Assessment of the prevalence of HCA derivatives in different living organisms and elucidation of their metabolism, including in human organs and tissues.
4. Expansion of the spectrum of amyloidogenic proteins, the pathological transformation of which is affected by HCA derivatives.
5. Development of targeted preparations for the prevention and treatment of various amyloid neurodegenerative diseases based on synthetic and natural derivatives of HCA.

## Figures and Tables

**Table 1:** Influence of hydroxycinnamic acid derivatives and structurally similar compounds (e.g., phenylacetic and benzoic acid derivatives) on the amyloid transformation of α-synuclein.

Name	Formula	Influence on Amyloid Transformation of α-Synuclein
Ferulic acid ^1^	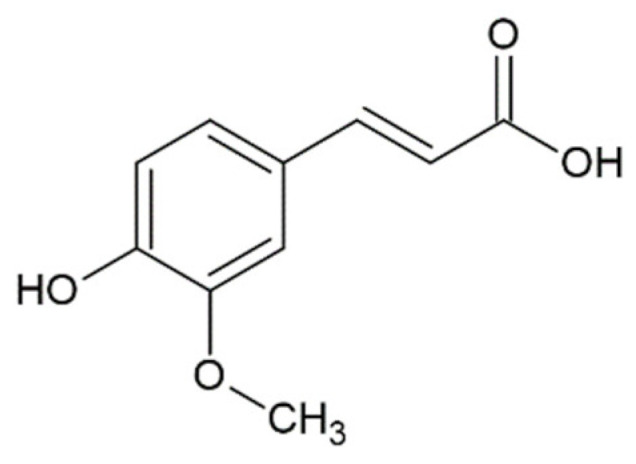	inhibition of fibrillation IC_50_ = 0.8 µM [161]; IC_50_ = 13 µM [118]
Gallic acid	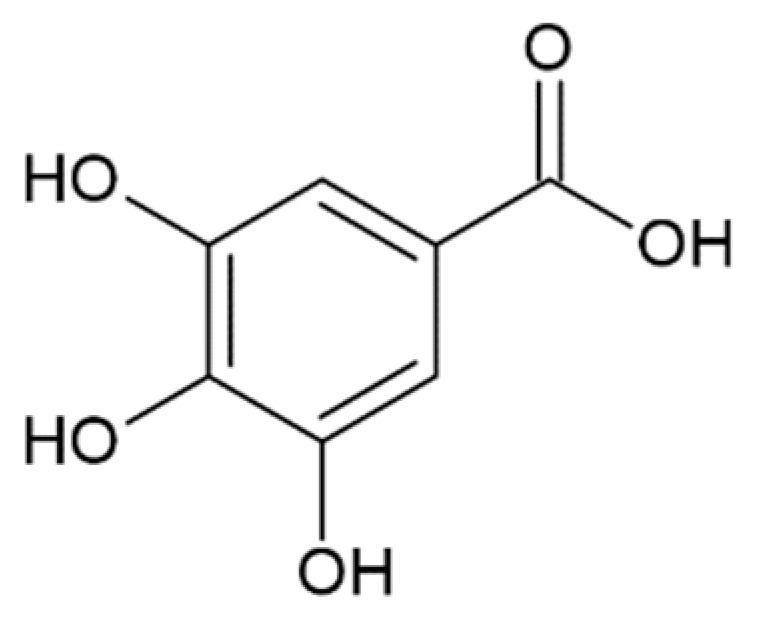	complete inhibition of fibrillation of A53T α-synuclein by 40 µM (monomer: gallic acid 1:2) [172] almost complete inhibition of fibrillation by 100 µM (monomer: gallic acid 1:1) [127] formation of soluble non-toxic oligomers with no β-structure, stable in denaturing conditions; monomer: gallic acid 1:4 - inhibition of oligomerization monomer: gallic acid 1:2 and 1:1—stabilization of oligomers [173]
Caffeic acid ^1^	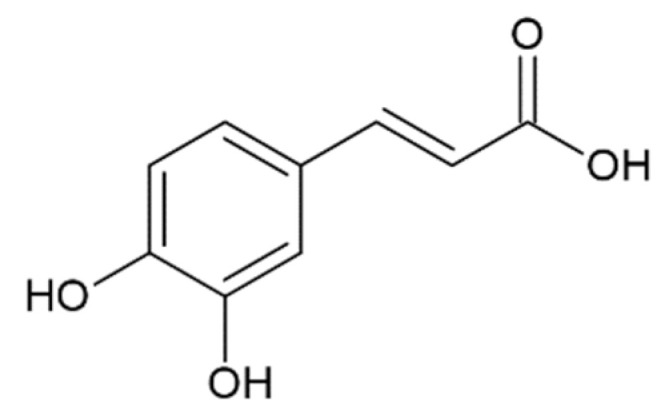	complete inhibition of fibrillation by 50-100 µM (monomer: caffeic acid 1:0.5 and 1:1) [127] active against an antidepressant escitalopram-induced fibrillogenesis of human a-synuclein: 60 µM (monomer: caffeic acid: escitalopram 1.2:1:1.4) warranted ~85% decrease in ThT fluorescence [129] no interaction with monomer [173] transient interactions with N- and C-terminal domains of monomer [172]
3-methoxy-4- acetamidoxycinnamic acid ^1^	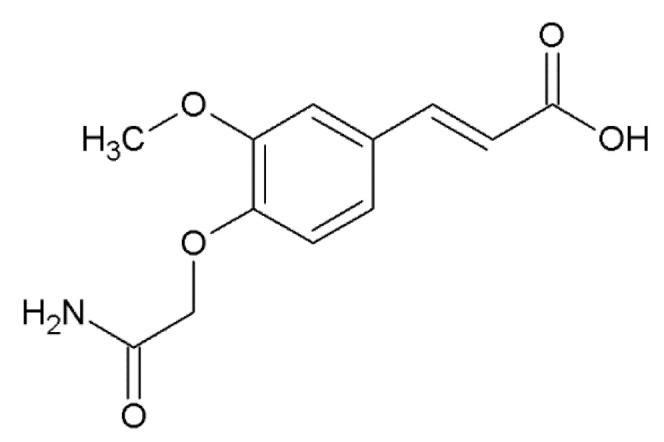	inhibition of fibrillation IC_50_ = 50 µM [118]
3,4-dihydroxyphenylacetic acid (DOPAC)	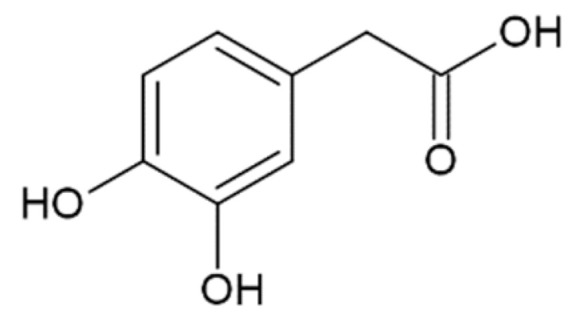	complete inhibition of fibrillation by 100 µM (monomer: DOPAC 1:1,4); formation of unstable α-synuclein oligomers [175] binding to the N-terminus of monomer [175]
Homogentisic acid	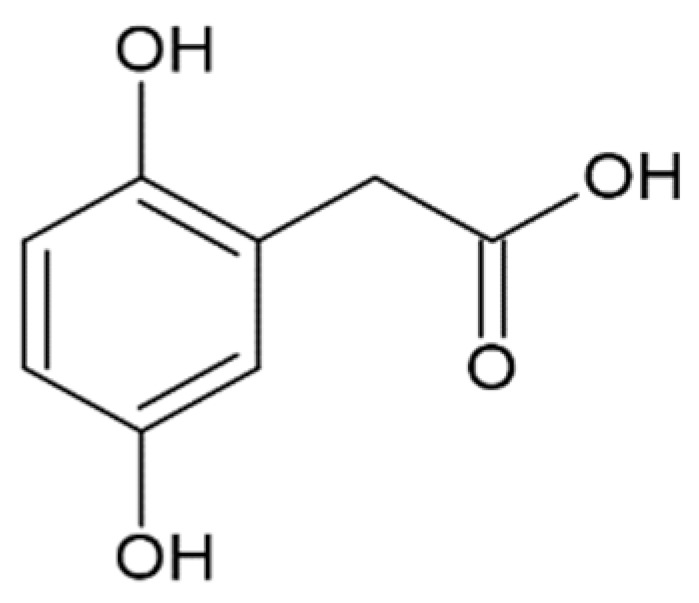	substantial inhibition of α-Syn fibrillation by ThT assay (data not shown) [175]
Pyrogallol	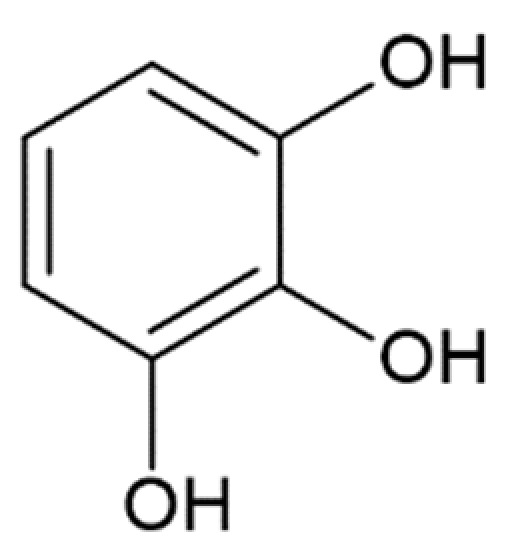	complete inhibition of fibrillation by 50-100 µM (monomer: pyrogallol 1:0.5 and 1:1) [127]
Protocatechuic acid	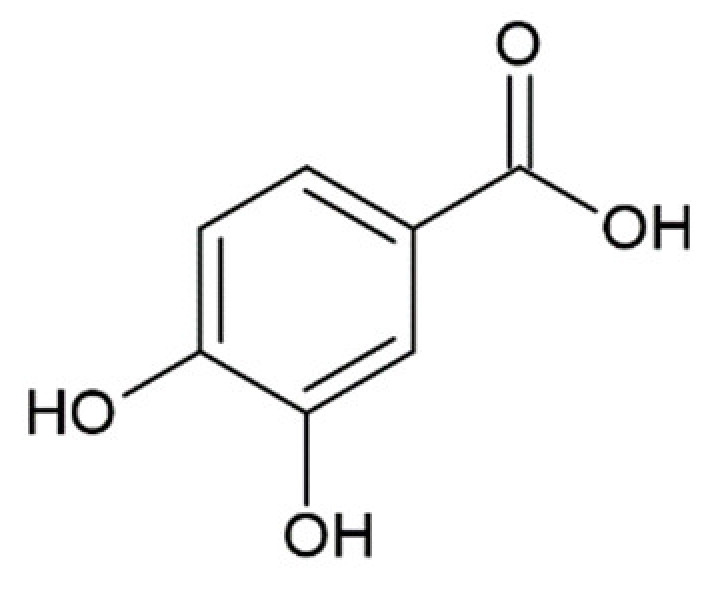	inhibition of fibrillation by 70% at 100 µM (monomer: protocatechuic acid 1:1.4) [174]
Hydroxytyrozol	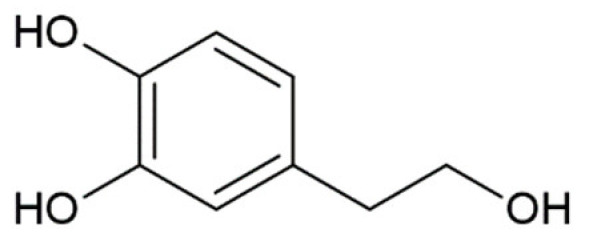	inhibition of fibrillation by 70% at 100 µM (monomer: hydroxytyrozol 1:1.4) [174]
2,4,6-trihydroxybenzoic acid	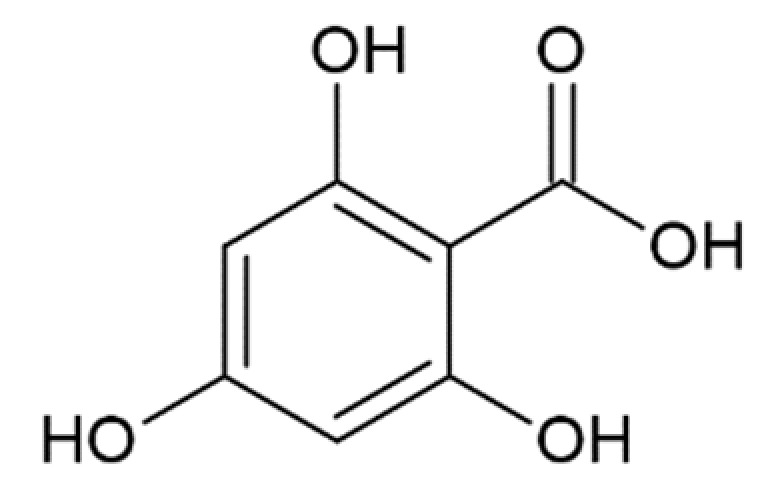	inhibition of fibrillation by ~72% at 100 µM, (monomer:2,4,6-trihydroxybenzoic acid 1:4) [173]
3,4-dihydroxybenzoic acid	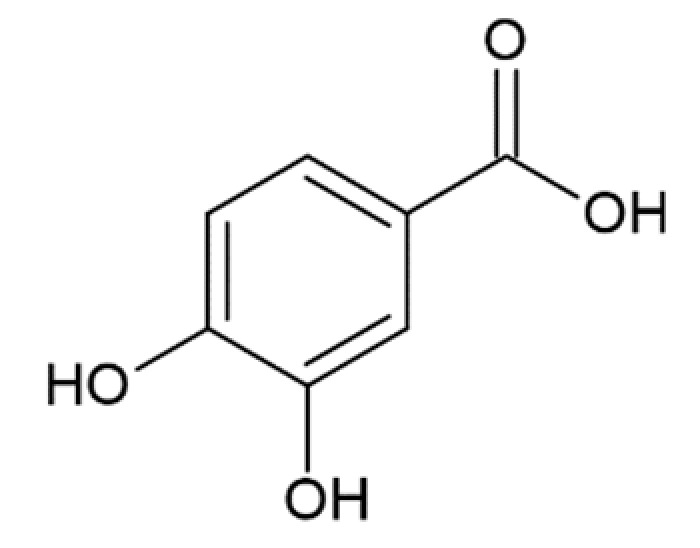	inhibition of fibrillation by ~60% at 100 µM, (monomer:3,4-dihydroxybenzoic acid 1:4) [173]
3,4-dimethoxycinnamic acid (3,4-DMCA)	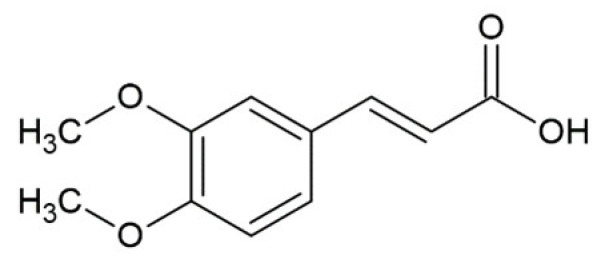	substantial inhibition (~90%) of α-Syn fibrillation by 280 µM (monomer: 3,4-DMCA 1:10), IC_50_ 251 µM [118]
O-methyl-dehydrozingerone^1^	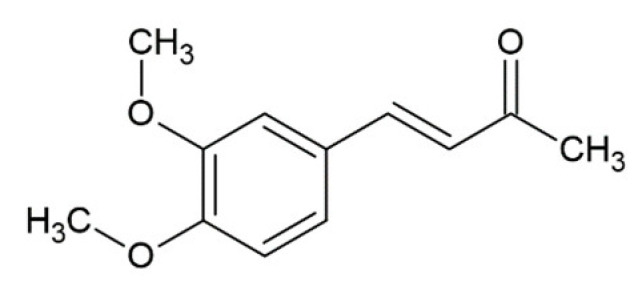	inhibition of fibrillation by ~30% at 250 µM, (monomer: O-methyl-dehydrozingerone 1:2.5), Congo red assay [155]
2,6-dihydroxybenzoic acid	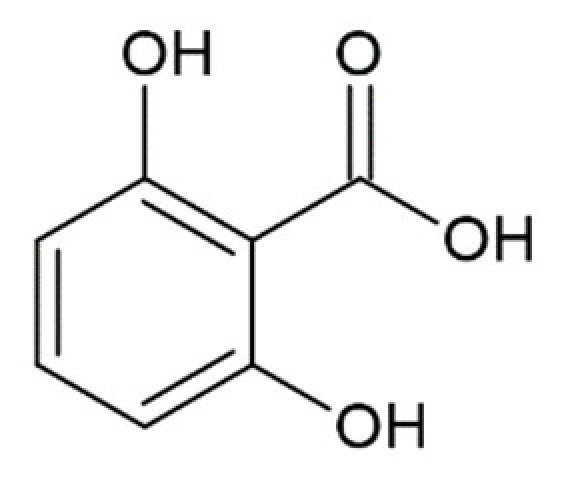	inhibition of fibrillation by ~30% at 100 µM, (monomer: 2,6-dihydroxybenzoic acid 1:4) [173]
4-hydroxybenzoic acid	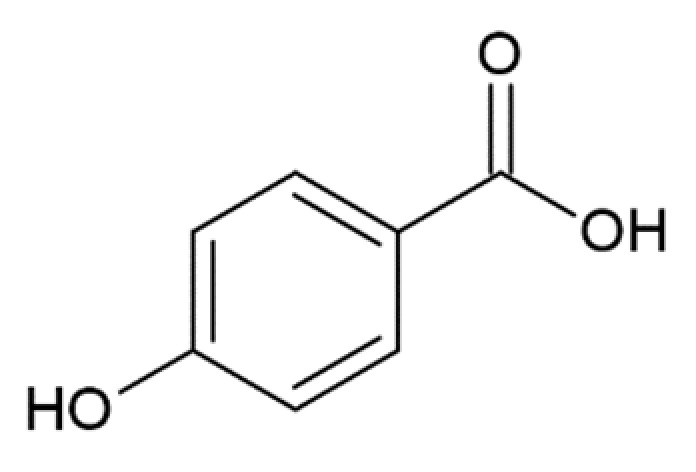	inhibition of fibrillation by ~30% at 100 µM, (monomer: benzoic acid 1:4) [173]
Benzoic acid	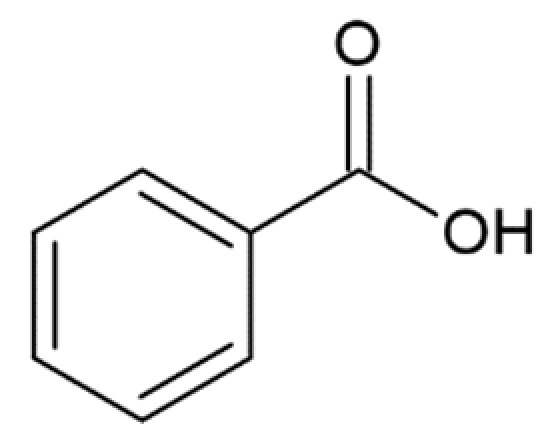	inhibition of fibrillation by ~5% at 100 µM, (monomer: benzoic acid 1:4) [173]
2-hydroxybenzoic acid (salicylic acid)	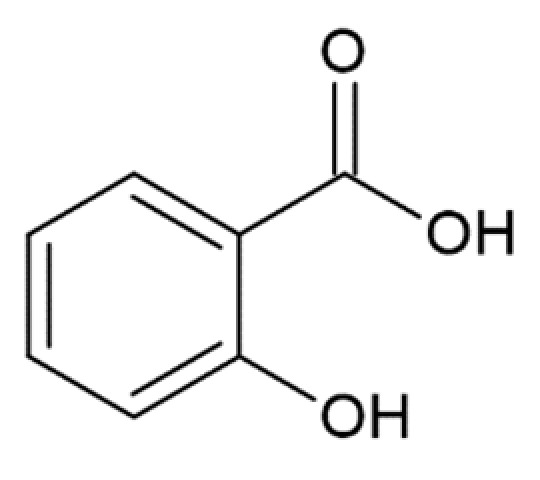	no anti-amyloid aggregation activity [173]
3,5-dihydroxybenzoic acid	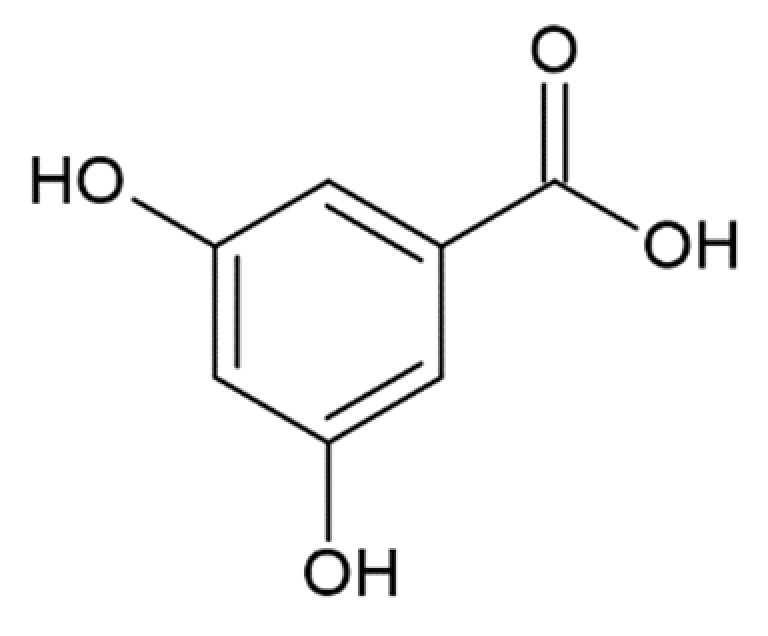	no anti-amyloid aggregation activity [173]
3-hydroxybenzoic acid	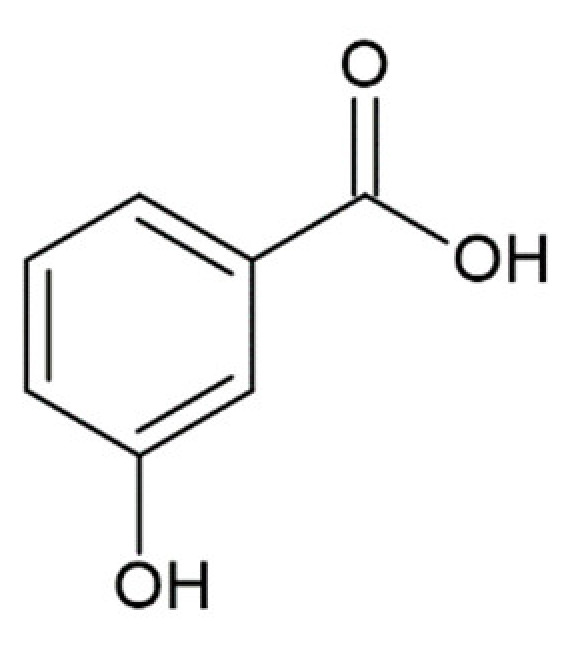	no anti-amyloid aggregation activity [173]

^1^ Hydroxycinnamic acid derivatives.
